# When Vomiting Isn’t Just a Bug: Unmasking Two Rare Causes of Pediatric Dysphagia

**DOI:** 10.7759/cureus.83451

**Published:** 2025-05-04

**Authors:** Noora AlSuwaidi, Bachir Farzat, Imad Hannoun

**Affiliations:** 1 Department of Emergency Medicine, Al Jalila Children's Specialty Hospital, Dubai, ARE

**Keywords:** angelman syndrome, barium swallow, chronic vomiting, double aortic arch, dysphagia, esophageal obstruction, pediatric achalasia, vascular ring

## Abstract

Pediatric vomiting and feeding difficulties are common presentations in both emergency and outpatient settings. Although these symptoms are often attributed to benign causes such as infections or gastroenteritis, structural and functional esophageal disorders must also be considered. This report describes two diagnostically challenging cases of esophageal obstruction in children. The first involves a 10-year-old boy with Angelman syndrome, ultimately diagnosed with a double aortic arch causing extrinsic esophageal compression. The second case features a previously healthy 13-year-old with chronic vomiting, later identified as having primary achalasia. These cases highlight the importance of maintaining a broad differential diagnosis and employing targeted imaging to prevent delays in diagnosis.

## Introduction

Vomiting and feeding difficulties are among the most common presenting complaints in pediatric practice. While these symptoms are often attributed to benign, self-limiting conditions such as viral gastroenteritis, clinicians must remain alert to more insidious and diagnostically challenging causes. Structural anomalies like vascular rings [1-3] and functional disorders such as achalasia [4-7] can closely mimic more common gastrointestinal conditions, frequently leading to significant diagnostic delays.

Pediatric achalasia is an uncommon motility disorder that often presents with subtle, nonspecific symptoms that are easily mistaken for more prevalent conditions like gastroesophageal reflux. Similarly, vascular rings, including double aortic arch or aberrant subclavian artery, may present with chronic vomiting, feeding intolerance, or recurrent respiratory symptoms, and are typically diagnosed only after a careful and systematic evaluation.

In this manuscript, we present two illustrative cases that highlight the diagnostic complexity associated with esophageal achalasia and vascular ring anomalies in children. Although these conditions are well documented in the literature, the cases emphasize the importance of maintaining a broad differential diagnosis when evaluating persistent vomiting and feeding issues. Through these reports, we aim to share practical clinical insights and raise awareness of these uncommon yet clinically significant conditions to support timely and accurate diagnosis in similar presentations.

## Case presentation

Case 1

In March 2025, a 10-year-old boy with a known diagnosis of Angelman syndrome was evaluated at the Pediatric Emergency Department of Al Jalila Children’s Specialty Hospital in Dubai, United Arab Emirates. He presented with a three-day history of progressively noisy breathing, accompanied by a two-day history of vomiting solids, while still tolerating liquids. Given the acute onset and his underlying neurodevelopmental condition, the initial differential diagnosis prioritized foreign body ingestion.

**Figure 1:**
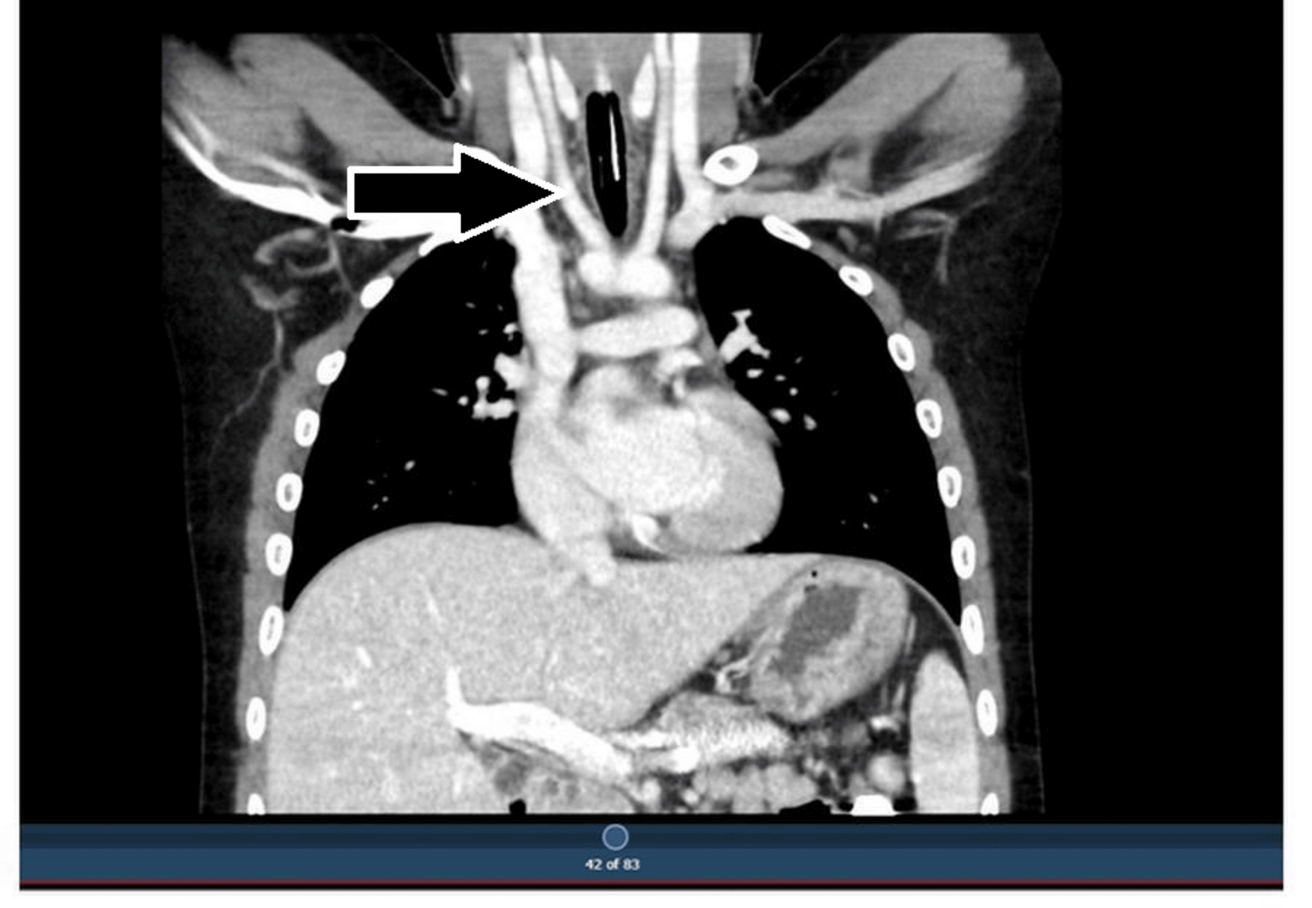
Coronal CT angiography showing a double aortic arch encircling the trachea and esophagus, leading to posterior esophageal compression

Clinical examination, including respiratory and abdominal assessments, was unremarkable. Baseline laboratory investigations were within normal limits (Table 1). A lateral neck radiograph revealed a soft tissue shadow in the prevertebral space at the level of C6-C7. Based on these findings, a barium swallow study was performed, which demonstrated an extrinsic posterior indentation of the esophagus. CT angiography subsequently confirmed the presence of a double aortic arch compressing the posterior esophageal wall (Figure 1) [3], establishing the diagnosis of a vascular ring anomaly.

**Table 1:** Laboratory values for Case 1

Test	Result	Reference range
WBC	6.9	4.5-13.5 × 10⁹/L
Hemoglobin	13.9	11.5-15.5 g/dL
Platelets	229	150-400 × 10⁹/L
Lipase	21	0-60 U/L
Amylase	31	28-100 U/L
Sodium	139	135-145 mmol/L
Potassium	4	3.5-5.0 mmol/L
Bicarbonate	19	20-28 mmol/L
Creatinine	0.58	0.5-1.0 mg/dL
Urea	29	2.5-7.5 mmol/L
Liver function tests	Normal	Normal

Case 2

Another case seen in March 2025 involved a 13-year-old previously healthy male who was referred to the Pediatric Emergency Department at Al Jalila Children’s Specialty Hospital in Dubai, United Arab Emirates, with a one-month history of persistent, non-bilious vomiting. He had undergone multiple outpatient evaluations, none of which yielded a definitive diagnosis. On arrival, his vital signs were stable, and physical examination was unremarkable. Laboratory investigations, including a metabolic panel and inflammatory markers, were within normal limits (Table 2).

**Table 2:** Laboratory values for Case 2

Test	Result	Reference range
WBC	7.9	4.5-13.5 × 10⁹/L
Hemoglobin	12.4	11.5-15.5 g/dL
Platelets	272	150-400 × 10⁹/L
Sodium	136	135-145 mmol/L
Potassium	4.2	3.5-5.0 mmol/L
Bicarbonate	19	20-28 mmol/L
Urea	21	2.5-7.5 mmol/L
Glucose	89	70-110 mg/dL
Liver function tests	Normal	Normal

**Figure 2:**
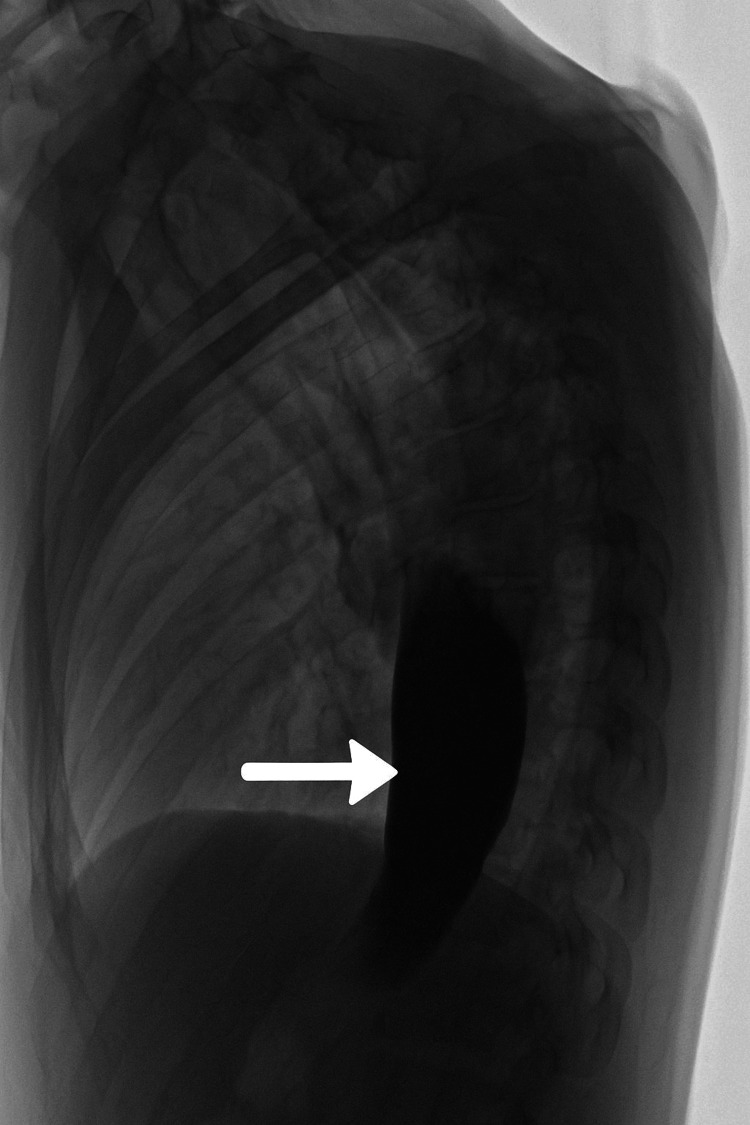
Lateral view from a barium swallow showing smooth tapering at the gastroesophageal junction, consistent with the “bird’s beak” sign of achalasia

Plain abdominal radiography revealed a dilated esophagus, raising suspicion for an underlying motility disorder. A barium swallow study showed a characteristic smooth tapering at the gastroesophageal junction, with delayed contrast transit that improved with the administration of water. These radiological findings were consistent with primary achalasia (Figure 2) [4-7].

## Discussion

Both double aortic arch and achalasia are rare but significant causes of esophageal obstruction in children [1-7]. Although they stem from fundamentally different pathologies - one congenital and structural, the other functional and neuromuscular - their overlapping symptoms can complicate the diagnosis.

Double aortic arch is the most common type of complete vascular ring, accounting for approximately 42% of all symptomatic vascular rings in children [1,2]. It results from the persistence of both fourth aortic arches during fetal development, forming a ring that compresses the trachea and esophagus. The incidence of symptomatic vascular rings is estimated to range from 1 in 10,000 to 1 in 50,000 live births. These anomalies often present in infancy or early childhood with feeding difficulties, stridor, or recurrent respiratory infections. CT angiography has become the preferred imaging modality for diagnosis due to its ability to clearly delineate vascular anatomy [3].

In contrast, achalasia is a primary motility disorder characterized by failure of the lower esophageal sphincter to relax and the absence of esophageal peristalsis. It is extremely rare in pediatrics, with an estimated incidence of 0.1 to 0.18 per 100,000 children per year [5]. Pediatric patients typically present with vomiting, weight loss, and dysphagia. A barium swallow classically shows a “bird’s beak” appearance of the distal esophagus, while esophageal manometry is the gold standard for diagnosis [4,6,7]. However, access to pediatric manometry may be limited in many centers.

These two cases highlight the importance of maintaining a broad differential diagnosis in children presenting with persistent vomiting or dysphagia, especially in those with neurodevelopmental disorders, where communication challenges can obscure classic symptoms. A high index of suspicion, coupled with early use of targeted imaging like barium swallow studies and CT angiography, can significantly aid in identifying esophageal abnormalities. Barium swallow remains a valuable diagnostic tool, particularly when endoscopy or manometry are not readily available. Additionally, incorporating cross-sectional imaging early in the diagnostic workup is crucial when symptoms persist despite an unremarkable initial evaluation. A systematic and vigilant approach to assessment allows for timely diagnosis and intervention, ultimately improving clinical outcomes and reducing the risk of long-term morbidity.

## Conclusions

Persistent vomiting and dysphagia in children should prompt consideration of rare structural and functional esophageal disorders, such as vascular rings and achalasia, particularly when standard evaluations yield inconclusive results. Early use of targeted imaging and maintaining a high index of suspicion are critical to ensuring timely diagnosis and effective management.
